# Impact of acetabular reaming depth on reconstruction of rotation center in primary total hip arthroplasty

**DOI:** 10.1186/s12891-018-2336-8

**Published:** 2018-11-30

**Authors:** Pu Shao, Zhizhou Li, Modi Yang, Yuzhuo Wang, Te Liu, Yuhui Yang, Lian Duan, Jinlan Jiang, Jianlin Zuo

**Affiliations:** 1 Department of Orthopedics, China-Japan Union Hospital of Jilin University, 126 xiantai street, Changchun, 130033 People’s Republic of China; 2 Scientific Research Centre of China-Japan Union Hospital of Jilin University, 126 xiantai street, Changchun, 130033 People’s Republic of China; 3 Department of Orthodontics, School and Hospital of Stomatology, Jilin University, Changchun, 130021 People’s Republic of China

**Keywords:** Total hip arthroplasty, Rotation center, Acetabulum, Reaming depth, Teardrop

## Abstract

**Purpose:**

To study the impact of acetabular reaming depth on reconstruction of rotation center (RC) in unilateral primary total hip arthroplasty (UPTHA) and guide individualized preoperative design.

**Methods:**

200 postoperative standard bilateral hip anteroposterior radiographs after UPTHA were included, which were collected from January, 2013 to June, 2017 in our hospital. Osteonecrosis of femoral head was the only diagnosis in this cohort. The parameters were measured on the anteropoterior radiographs by using RadiAnt DICOM viewer.

**Results:**

The average of the thickness of the teardrop is about 6.13 ± 1.42 mm. The parameter a (the difference value of the distance of bilateral RC and midline) was positively correlated with the parameter e (the acetabular reaming depth), and the Pearson correlation coefficient was 0.49 when *P* = 0.05. Furthermore, the value of parameter (e) was 8.25 mm when a2 (the distance from the center of the prosthesis femoral head to the vertical line across the midpoint of pubic symphysis) equaled a1 (the distance from RC of the healthy femoral head to the vertical line across the midpoint of pubic symphysis).

**Conclusions:**

The reaming depth of the acetabulum could influence the reconstruction of RC during UPTHA. When the medial margin of the cup was placed about 2 mm to the lateral border of the ipsilateral teardrop (the bottom of the ovum), the rotation center would be accurately restored.

## Introduction

It is now acknowledged that total hip arthroplasty (THA) is one of the most successful procedures in the field of orthopeadics, which is widely applied to the treatment of many hip joint diseases resulted in dysfunction of hip joint [1]. Maximization of the prosthesis life is the most significant issue for orthopedic surgeons [2].

Among the influencing factors for successful restoration of joint, accurate biomechanical reconstruction of the femur and acetabulum is essential for satisfactory function achievement [3–5]. In order to reduce the occurrence of many postoperative complications it is necessary to Reproduce, as close as possible, normal or near-normal mechanics. One of the most important steps in achieving this is to transfer the hip joint RC into the true acetabulum [6–8]. The native acetabulum is subhemispherical in most cases, but the acetabular components used for THA are hemispherical, which inadvertently leads to displacement of RC when the acetabular component is fully implanted [9, 10]. Hence, it is very vital for hip replacement to reconstruct RC of femoral head prosthesis in the anatomical position of the acetabulum side. Moreover, the RC of hip is also pivotal for recovery of muscle function [4, 11–15], joint stability [16–19], and hip prostheses longevity [20–23]. If RC of femoral head prosthesis is not placed at the anatomical position, it may also lead to patients with unequal limb length, unequal femoral eccentricity, etc. Over time it will cause the muscles to lose their original function, the joints are unstable, and eventually the loosening of the prosthesis.

So, the depth of acetabular prosthesis implantation becomes a main factor infecting the position of RC. Despite advances in surgery technique, the landmark of acetabular reaming depth for anatomic restoration of RC remains uncertain. In this paper, standard bilateral hip anteroposterior radiographs after unilateral primary THA (UPTHA) were used to clarify the accurate acetabular reaming depth for anatomically reconstructing RC of hip joint during THA.

## Methods

### General information

In this study, 200 standard bilateral hip anteroposterior radiographs after UPTHA were collected from January 2013 to June 2017 in our hospital. As shown in Table 1, there were 116 males and 84 females in all subjects. Among them, there were 97 patients on the left side and 103 patients on the right side. The average age was 58.1 years (from 24 to 80 years). The inclusion criterions applied was as follow: Patients with unilateral osteonecrosis of femoral head while the contralateral hip joint was normal. The exclusion criterions used were as follow: 1 Patients with unilateral or bilateral acetabular or femoral developmental dysplasia; 2. Patients with unilateral or bilateral hip joint fracture history.

**Table 1:** General information of the 200 patients

Item	Classification	Number	Percentage
Sexuality	Male	116	58%
	Female	84	42%
Age	<40	12	6%
	40–60	100	50%
	>60	88	44%
Operation Side	Left	97	48.5%
	Right	103	51.5%

### X-ray and measurements

The standard bilateral hip anteroposterior radiographs were gathered after UPTHA. The criteria for taking the radiographs were as follow: The patients were supine on the photography table, with median sagittal plane coincided with the cassette midline, the lower limbs were fully extended, and were placed in internal rotation about 15°. The X-ray filter was used. The tube distance was 100 cm. The center of the beam was focused on the point 3 cm below the pubic symphysis midpoint, and the beam was vertically injected into the cassette. The position was controlled so as to make the bilateral obturator foramens isometrical and the tip of coccyx locating at the level and in the center of the pubic symphysis. The longitudinal axes of bilateral femurs were parallel to each other and to the longitudinal central axis of the pelvis.

**Fig. 1:**
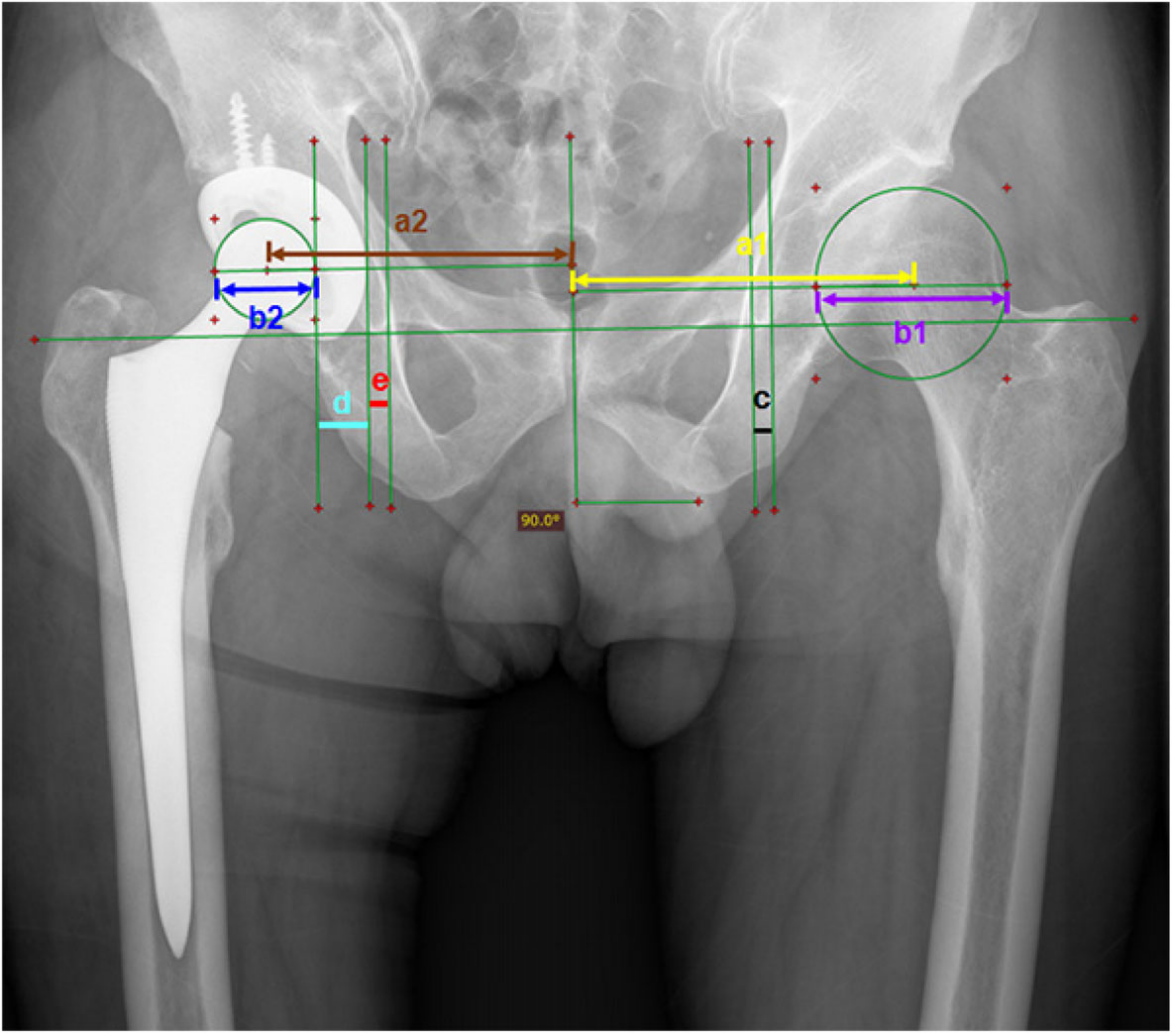
Postoperative measurement: the distance between the center of rotation of the healthy femoral head to the vertical line across the midpoint of pubic symphysis (a1); the distance between the center of the prosthesis femoral head to the vertical line across the midpoint of pubic symphysis (a2); the diameter of the healthy femoral head (b1); the diameter of the prosthesis femoral head (b2); the thickness of the teardrop(c); the distance between the medial edge of prosthesis femoral head and the medial edge of the acetabular cup (d); the distance between the medial edge of the acetabular cup and the medial edge of teardrop (e)

As shown in Fig. 1, RadiAnt DICOM viewer (version 1.9.16, 32-bit, developed by Medixant Company in Poland in 2009) was applied to measure the following parameters on the above mentioned postoperative standard bilateral hip anteroposterior radiographs. First a transverse line passing through the lower edges of bilateral teardrops were drawn. Then the following measurements were made: a1. The distance from RC of the healthy femoral head to the vertical line across the midpoint of pubic symphysis; a2. The distance from the center of the prosthesis femoral head to the vertical line across the midpoint of pubic symphysis; b1. The diameter of the healthy femoral head; b2. The diameter of the femoral head prosthesis; c. The thickness of the teardrop on the contralateral side; d. The distance between the vertical lines tangent to the medial margins of the femoral head prosthesis and the acetabular prosthesis; e. The distance between the vertical lines tangent to the medial margins of the acetabular prosthesis and the medial border of the ipsilateral teardrop, which was defined as the horizontal reaming depth.

### Correction and standardization of the measurements

After the measurement of the above parameters, the value correction was done. Parameter b2, the diameter of the femoral head prosthesis, was a known data, which could be retrieved from the patient chart. So, the ratio of the true value and the measured value of the femoral head prosthesis could be obtained in each case, which was named as correction coefficient. By multiplying each patient’s data by the corresponding correction coefficient, the true value of each parameter could be obtained in each case. Standardization of the measured data was subsequently carried out in order to make the results comparable among the 200 patients with different heights and weights. After the measurements and corrections, the ratio of the diameter of the largest to the diameter of every other femoral head, called standardized coefficient, was obtained. Then, by multiplying the true value of each parameter with the standardized coefficient the standard value of each parameter was acquired.

### Statistical analysis

SPSS 22.0 was applied for statistical analysis. Pearson correlation analysis was used to analyze the correlation between the parameter (a) (a = a2 – a1) and the parameter (e), and the pattern of relevant scatter plot was drawn. The illustration of relationship of parameter (e) and parameter (a) was further progressed by regression analysis.

## Results

**Fig. 2:**
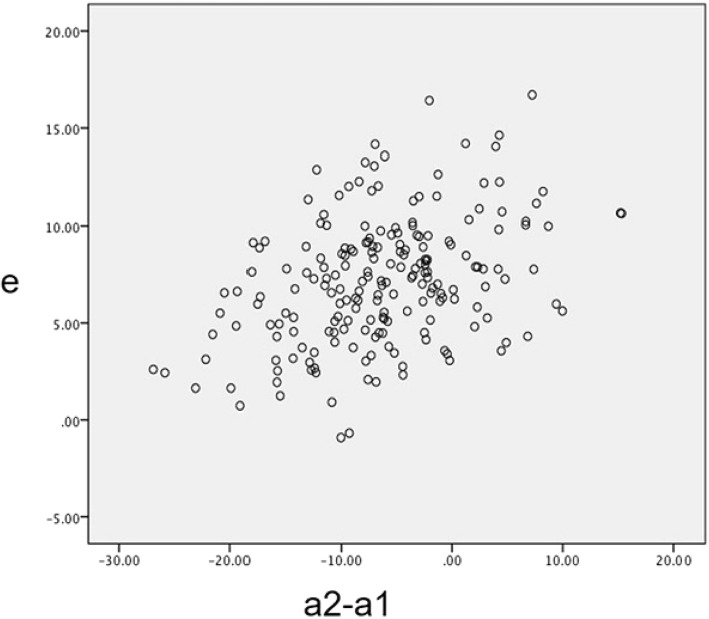
The pattern of linear correlation scatter plot of the reaming depth (e) and the difference value of the distance of bilateral RC and midline (a2 - a1)

The diameter of the largest femoral head on the healthy side was 55.5mm. After the Pearson correlation analysis is performed for parameter a (the difference value of the distance of bilateral RC and midline) and parameter e(the distance between the vertical lines tangent to the medial margins of the acetabular prosthesis and the medial border of the ipsilateral teardrop), it can be concluded that the parameter (a) was positively correlated with the parameter (e), and the Pearson correlation coefficient was 0.49, when *P* = 0.05. That is to say the parameter (e) is increased with an increase in the parameter (a). As shown in Fig. 2, the pattern of the relevant scatter plot was suggested that the parameter (a) was positively correlated with the parameter (e). In addition, the regression analysis was further shown that the coefficients of the independent variables passed the statistical test at a confidence level of 0.05. The regression equation was finally established as follow:

$$ \mathrm{e}=8.25+0.17\times \left(\mathrm{a}2\hbox{-} \mathrm{a}1\right) $$

Hence, when the RC of the operated hip was restored anatomically in the horizontal direction, that is (a) = 0, the distance between the medial tangent line of the acetabular prosthesis and that of the lateral border of the ipsilateral teardrop, that is e, equaled 8.25 mm. This result was got at the condition that the diameter of the femoral head was 55.5 mm. Because of the medial margin of the teardrop cannot be seen during operation, we measured the thickness of teardrop, which is 6.13 mm ± 1.42 mm. Then we drew the conclusion that when the medial margin of the cup was about 2 mm (8.25 mm minus 6.13 mm equals about 2 mm) lateral to the lateral margin of the ipsilateral teardrop the rotation center of the operated hip would be anatomically restored.

## Discussion

In all precedures of preoperative plan for THA, the anatomical reconstruction of acetabular RC is a very important target [24, 25]. The position of RC of the operated hip was the most important factor for the stress and function of the soft tissue around the hip joint. And in the anatomical reconstruction of the horizontal of the RC, the horizontal reaming depth of the acetabulum plays a very important role. The ideal position of acetabular prosthesis mainly depended on the femoral head size and the neck diameter. For the long-term service life of the prosthesis, the acetabulum should be placed in its anatomical position to normally inclusion of the acetabular prosthesis and restore the length of the limb [26]. In the existing literature reports, no one has studied the goal of the ideal depth of acetabular reaming. The acetabular reaming depth affects the position of the RC in horizon direction. If the reaming depth is too deep or too shallow, it may affect the survivorship of the prosthesis by changing the force torque of the reconstructed hip. There is no uniform standard for acetabular prosthesis depth in THA, so far. In this paper, standard bilateral hip anteroposterior radiographs after unilateral primary THA (UPTHA) were used to clarify the accurate acetabular reaming depth for anatomically reconstructing RC of hip joint during THA. And it will have a great potential in individualized preoperative plan of THA.

In this paper, the distance (a2) between the RC of operated and midline can be divided into three parts: the radius of femoral head component (half of b2), the thickness of acetabular cup (d) (including metal acetabular cup and high molecular polyethylene liner), and the reaming depth (e) (the distance between the medial tangent line of the acetabular component and that of the medial border of the ipsilateral teardrop) in the postoperative anteroposterior radiographs of THA. Because the radius of femoral head component and the thickness of acetabular component were two constant values, after the data correction and standardization, the reaming depth (e) became the only one variable affecting the reconstruction of RC. In this paper, we did correction to exclude influence of the magnification of the radiographs by calculating the ratio of the true value of the femoral head prosthesis (was a known data, which could be retrieved from the patient chart.) and the measured value of the femoral head prosthesis. Then we performed standardization to exclude influence of bone size on the measurements. We used diameter of the largest healthy femoral head (55.5 mm) in the 200 patients as the standard, the measurements of other patients were then multiplied by the ratio of the diameter of the corresponding femoral head to that of the largest femoral head (55.5 mm). By balancing different sizes of bone from this cohort of patients, we could analyze if there were linear correlations between the parameters. According to statistical analysis, it was suggested that the reaming depth (e) in horizon direction would be 8.25 mm under the condition that the diameter of healthy femoral head was 55.5 mm and RC was restored anatomically (a1 = a2).

It is well known that the medial margin of teardrops is visible in the radiographs imaging but invisible in the surgery procedure. But the lateral border of the teardrop is visible in the surgery procedure. It is the bottom of the ovum. Therefore, we further measured the thickness of the teardrop(c), so as to obtain the best distance between the vertical lines tangent to the medial margins of the acetabular cup and the lateral border of the teardrop. Then we drew the conclusion that when the medial margin of the cup was about 2 mm (8.25 mm minus 6.13 mm equals about 2 mm) lateral to the lateral margin of the ipsilateral teardrop the rotation center of the operated hip would be anatomically restored. Consequently, in this work, a new standardized method of restoration of RC was provided, and it will have a great potential in individualized preoperative plan of THA.

The limitation of this study is that the thickness of the cup prosthesis varies a little bit among different brands, and the thickness of the linear changes with the size of the cup. And we did not record height and weight of the patients in this cohort. But the influences of the former two factors were already minimized by standardization of the data.

## Conclusion

We found that the reaming depth of the acetabulum influence the reconstruction of RC during THA. In the procedures of individualized preoperative plan, the distance between the medial tangent line of the acetabular prosthesis and that of the medial border of the ipsilateral teardrop, e = 8.25 mm, when the diameter of the healthy femoral head was 55.5 mm. Because of the average thickness of the normal teardrop(c) is 6.13 ± 1.42 mm, we can conclude that when the medial margin of the cup was placed about 2 mm to the lateral border of the ipsilateral teardrop (the bottom of the ovum), the rotation center would be accurately restored.

## Abbreviations

RC: Rotation center
THA: Total hip arthroplasty
UPTHA: Unilateral primary total hip arthroplasty
